# Oxidative Stress and the Use of Antioxidants in Stroke

**DOI:** 10.3390/antiox3030472

**Published:** 2014-07-03

**Authors:** Rachel Shirley, Emily N. J. Ord, Lorraine M. Work

**Affiliations:** Institute of Cardiovascular & Medical Sciences, College of Medical, Veterinary & Life Sciences, University of Glasgow, BHF GCRC, 126 University Place, Glasgow G12 8TA, UK; E-Mails: Rachel.Shirley@glasgow.ac.uk (R.S.); Emily.Ord@glasgow.ac.uk (E.N.J.O.)

**Keywords:** anti-oxidant, stroke, oxidative stress

## Abstract

Transient or permanent interruption of cerebral blood flow by occlusion of a cerebral artery gives rise to an ischaemic stroke leading to irreversible damage or dysfunction to the cells within the affected tissue along with permanent or reversible neurological deficit. Extensive research has identified excitotoxicity, oxidative stress, inflammation and cell death as key contributory pathways underlying lesion progression. The cornerstone of treatment for acute ischaemic stroke remains reperfusion therapy with recombinant tissue plasminogen activator (rt-PA). The downstream sequelae of events resulting from spontaneous or pharmacological reperfusion lead to an imbalance in the production of harmful reactive oxygen species (ROS) over endogenous anti-oxidant protection strategies. As such, anti-oxidant therapy has long been investigated as a means to reduce the extent of injury resulting from ischaemic stroke with varying degrees of success. Here we discuss the production and source of these ROS and the various strategies employed to modulate levels. These strategies broadly attempt to inhibit ROS production or increase scavenging or degradation of ROS. While early clinical studies have failed to translate success from bench to bedside, the combination of anti-oxidants with existing thrombolytics or novel neuroprotectants may represent an avenue worthy of clinical investigation. Clearly, there is a pressing need to identify new therapeutic alternatives for the vast majority of patients who are not eligible to receive rt-PA for this debilitating and devastating disease.

## 1. Introduction

In addition to being the second leading cause of death worldwide [1], stroke is also the leading cause of acquired adult disability [2]. Stroke therefore has a very large socioeconomic impact with one third of all stroke patients requiring permanent residential care, costing the NHS in the UK £3.8 billion per annum [3]. The only approved pharmacological intervention for stroke is intravenous administration of the thrombolytic, recombinant tissue plasminogen activator (rtPA), within 4.5 h of the onset of ischaemia [4]. This short therapeutic window results in only 2%–5% of all stroke patients receiving this intervention with successful reperfusion of the brain occurring in only 50% of that cohort [5], as such this is a disease with largely unmet clinical needs. Ischaemic stroke (as opposed to haemorrhagic stroke) results from an atherothrombotic or embolic blockage to a cerebral artery and accounts for ~80% of all stroke cases. Finding a new pharmacological treatment for stroke is incredibly complex for a number of reasons. The brain is a highly metabolically active organ that relies on constant oxygen and glucose supply from the circulation. The brain accounts for 2% of the total body but requires 20% O_2_ and 2% of the entire body’s glucose consumption, although it performs no mechanical work or external secretory activity [6]. Storage of energy and metabolites within the brain is extremely low and as such the brain is exceptionally sensitive to interruptions in blood flow [7]. Although the brain is protected from systemic toxins under normal physiological conditions by the blood-brain barrier (BBB), this is broken down during cerebral ischaemia allowing infiltration of inflammatory mediators and other potentially toxic molecules. Following the onset of ischaemia numerous pathways contribute to brain injury, there is extensive crosstalk between these deleterious pathways with them also, in the main, existing as positive feedback loops serving to amplify insult. In what is the most significant challenge of stroke treatment, the majority of damage occurs within minutes and the acute hours following cerebral ischaemia and as such, stroke treatment at present remains mainly preventative. Here, we will discuss the source and consequence of reactive oxygen species (ROS) imbalance following cerebral ischaemia/reperfusion before considering anti-oxidant strategies attempted clinically based on the pre-clinical evidence for each. What is clear is that while the contributory role of ROS in stroke cannot be disputed, the relative merit of anti-oxidant therapy alone has yet to be established and combined therapy may be where agents modulating the balance of ROS prove beneficial.

## 2. Sources of Reactive Oxygen Species (ROS)

**Figure 1:**
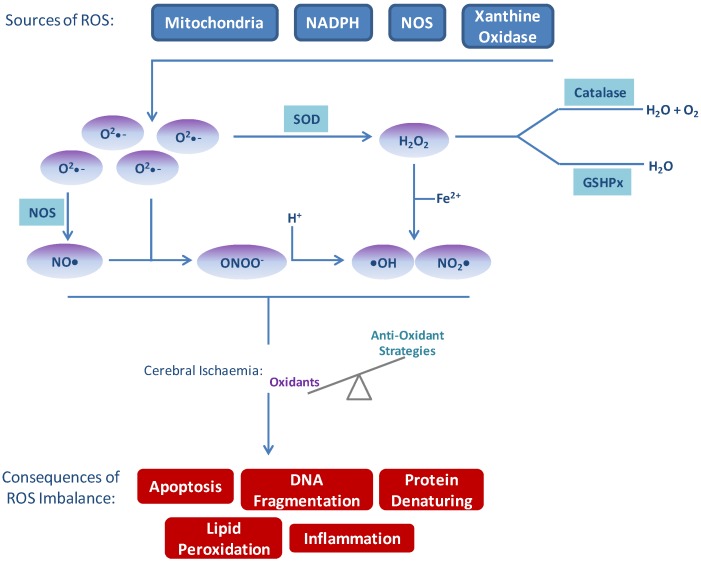
Sources of reactive oxygen species (ROS) and consequences of imbalance. Several sources of ROS exist endogenously which are balanced by natural anti-oxidant systems. In times of excess ROS production, such as following cerebral ischaemia/reperfusion, the balance of oxidants exceeds anti-oxidant protection. Excess ROS, in turn, result in apoptosis, inflammation, DNA damage, lipid peroxidation and protein denaturation—all of which contribute to and amplify signals leading to damage following stroke.

Oxidative stress as a result of excess production of ROS and/or impaired metabolism is a fundamental mechanism of cell damage following cerebral ischaemia, where ROS refers to molecular oxygen (•O_2_^−^) or its derivatives. Although oxidative stress has been implicated in numerous pathologies including cancer, atherosclerosis and neurodegenerative diseases, the brain is particularly sensitive to oxidative damage (reviewed in [8]). There are several reasons for this; the high consumption of oxygen under basal conditions, high concentrations of peroxidisable lipids, and high levels of iron that act as a pro-oxidant during stress. The primary sources of ROS in the brain are the mitochondrial respiratory chain (MRC), NAPDH oxidases, and xanthine oxidase [9,10,11] (Figure 1). Under normal cellular conditions, mitochondria produce superoxide as a by-product of their primary function—ATP generation by oxidative phosphorylation through the MRC. Superoxide produced in this way gets converted to hydrogen peroxide (H_2_O_2_) by superoxide dismutase (SOD) before leaving the mitochondria to act as an intracellular messenger, functioning in neuronal signalling in both the peripheral nervous system (PNS) and the central nervous system (CNS) [12]. In the ischaemic cell, O_2_ levels are depleted before glucose, favouring a switch to the glycolytic pathway of anaerobic ATP production [13]. The switch to glycolysis in the O_2_-depleted cell results in lactate acid and H^+^ build-up in the mitochondria and the subsequent reversal of the H^+^ uniporter on the mitochondrial membrane which causes excess cytosolic H^+^ accumulation and acidosis [14]. Acidosis contributes to oxidative stress by providing H^+^ for the conversion of •O_2_^−^ into H_2_O_2_ or the more reactive hydroxyl radical (•OH). In addition, in the O_2_-depleted cell the potent protein and lipid oxidant peroxynitrite (ONOO^−^) is formed by the reaction of nitric oxide (NO) and •O_2_^−^, rapidly exhausting the NO bioavailability. Activation of NMDA receptors (NMDARs) by glutamate also increases intracellular NO and subsequent ONOO^−^ production in the ATP depleted post-synaptic cell. Neuronal nitric oxide synthase (nNOS) is physically anchored to NMDARs and following activation and influx of Ca^2+^, Ca^2+^ binds calmodulin and rapidly activates nNOS generating NO [15]. NO reacts with superoxide anions (•O_2_^−^), produced by anaerobic metabolism of the energy-depleted mitochondria, forming ONOO^−^. ONOO^−^ mediates apoptosis through classic oxidative stress pathways described in a later section of this review (Section 3.1). In what is a well-documented paradigm of stroke, reperfusion of the previously ischaemic brain has severely deleterious cellular effects, including a large increase in ROS production [16,17]. The recovery of the MRC following return of cerebral blood flow causes a spike in the production of mitochondrial ROS. The reversal of complex I of the MRC and the subsequent overproduction of •O_2_^−^ is believed to be a significant contributor [18]. More recent studies have further demonstrated the importance of complex I, the entry point for electrons from NADH in the MRC, following ischaemic injury [19,20]. Pretreatment of neonatal mice with pyridaben to inhibit complex I resulted in reduced infarct volume determined 7 days after cerebral hypoxic-ischaemic injury [19]. Similarly, inhibition of complex I reactivation by *S*-nitrosation of a cysteine residue decreased ROS production, oxidative damage and tissue necrosis following cardiac ischaemia/reperfusion [20].

In addition to mitochondrial ROS production, nicotinamide adenine dinucleotide phosphate-oxidases (NOXs) are a significant source of ROS production following cerebral ischaemia and specifically during reperfusion injury [21]. The NOX family has seven members (NOX1, NOX2, NOX3, NOX4, NOX5, DUOX1, and DUOX2) with the isoforms NOX2, NOX3 and NOX4 predominantly expressed in the CNS. Under normal physiological conditions NOX enzymes function as membrane bound enzymes which generate ROS for biological functions such as blood pressure regulation, microbial killing and otoconia formation [22]. The catalysis of •O_2_^−^ occurs by one electron reduction of O_2_ using NADPH as an electron donor: 2O_2_ + NADPH → 2O_2_^−^ NAPD + H^+^. Under pathological conditions however, NOXs are significant contributors to pathological damage by oxidative stress from •O_2_^−^ overproduction and ROS imbalance. Although all seven NOX isoforms catalyse the reduction of molecular oxygen their mechanism of activation differs between isoforms. NOX2 is activated and simulated by phosphorylation-activated p47phox and by p67phox, in conjunction with activated Rac. NOX3 is activated by NOXO1 but is insensitive to additional stimulation by NOXA1 or activated Rac [23]. Activation of NOX4 has not been fully elucidated however it is thought to be controlled by transcriptional factors [24].

Xanthine oxidase (XO) functions to catalyse the oxidation of hypoxanthine to xanthine and oxidation of xanthine to uric acid. Under normoxic conditions XO exists as in interchangeable form of xanthine hydrogenase (XDH) [25]. During ischaemia cellular adenosine triphosphate (ATP) is catabolised to hypoxanthine which accumulates in the ischaemic tissue, and XDH is cleaved to the active XO. During the reperfusion phase, XO can then oxidise the reaction of hypoxanthine to xanthine and xanthine to uric acid, producing •O_2_^−^ and H_2_O_2_ [26].

## 3. Consequences of ROS Imbalance

The results of ROS imbalance are considerable and affect many processes within the brain parenchyma and all cells of the neurovascular unit (Figure 1). These events should not be considered as distinct from one another as considerable crosstalk and amplification occurs.

### 3.1. Apoptosis

ROS have a number of direct detrimental effects which result in cell damage and tissue destruction, such as: lipid peroxidation, protein denaturing, disruption of DNA and multiple cell signalling effects resulting in initiation of apoptosis [27,28,29]. Lipid peroxidation is the main mechanism by which ROS cause damage within the brain. Initiation of lipid peroxidation results in a positive-feedback loop of damage. The •OH initiates removal of one H^+^ from a polyunsaturated fatty acid resulting in formation of a lipid radical that reacts readily with O_2_ to form a lipid peroxyl radical and another lipid radical. The lipid radical by-product will further react with oxygen causing a positive feedback reaction. As an example of the positive-feedback loop of lipid peroxidation: excess ROS can activate phospholipase A_2_ (PLA_2_) which in turn releases and activates arachidonic acid (AA) and a by-product of the activation of AA is ROS. ROS act directly on lipids to ultimately produce aldehydes, dienals or alkanes, such as malondialdehyde (MDA) and 4-hydroxynonenal (4-HNE). The latter, 4-HNE induces apoptosis following cerebral ischaemia in neurons [30] and has been shown to be upregulated in a time-dependent manner in the ipsilateral striatum following focal ischaemia in rats [31]. Direct oxidative damage of DNA by ROS differs to endonuclease-mediated DNA fragmentation in that it occurs at earlier time-points following cerebral ischaemia and is potentially reversible. Oxidative damage to DNA occurs from direct attack of ROS on DNA, resulting in primarily DNA base damage and single-strand breaks (SSBs), observed in rodent models of cerebral ischaemia [32,33,34]. Although oxidative damage is potentially reversible, in cerebral ischaemia the multiple mechanisms that induce ROS ultimately generate fatal DNA damage to neuronal cells due to the ROS/antioxidant imbalance [33,35].

As aforementioned, a primary site for ROS generation following cerebral ischaemia is the mitochondria, where they exert their most detrimental role in initiation of cell death via cytochrome C (CytC) release [36,37]. Mitochondrial CytC release is in part controlled by the Bcl-2 family of proteins which are vitally involved in neuronal survival and programmed cell death. This gene family contains both pro-apoptotic and anti-apoptotic proteins and the anti-apoptotic members of the Bcl-2 family act by inhibiting the pro-apoptotic members. High [Ca^2+^]_i_ caused by excitotoxicty activates release of pro-apoptotic mediators from the mitochondria through activation of calpains [38] causing mitochondrial transition pore (MTP) opening. ROS can also mediate mitochondrial permeabilisation through activation of cytosolic phospholipase A_2_ (cPLA_2_). Activated cPLA_2_ triggers AA release by freeing it from an inhibiting phospholipid molecule through an enzymatic process. Activated AA has been shown to directly act on the MTP on mitochondria [39]. Release of pro-apoptotic CytC from the mitochondria results in initiation of the mitochondrial caspase-dependant intrinsic pathway of cell death. Following release, CytC forms a complex with cytosolic adapter protein (APAF-1) and caspase-9, known as the apoptosome, to mediate the activation of pro-caspase-9 in the presence of deoxy-ATP (dATP). Activated caspase-9 cleaves and activates caspase-3, which initiates apoptosis through DNA fragmentation by releasing the endonuclease caspase-activated DNase (CAD) from inhibition by cleaving its inhibitor ICAD [40,41]. CAD is a deoxyribose- and double-strand-specific enzyme [42] which preferentially cleaves the internucleosomal linker regions in chromatin [43].

ROS also mediate apoptosis through a direct interaction with nuclear factor κB (NF-κB) and subsequent activation of the MAPK/JNK pathway of cell death. NF-κB is known to be activated by the redox state of the cell in a number of disease pathologies and activation can be inhibited through use of antioxidants [44]. Additional downstream gene targets of NF-κB also include, NOS [45], cyclooxygenase-2 (COX-2) [46], matrix metalloproteinase-9 (MMP-9) [47], intracellular adhesion molecules (ICAMs) and cytokines [48] which are involved in a number of detrimental pathways of cerebral ischaemia such as, apoptosis, BBB breakdown, and inflammation. JNK activates mitochondrial-mediated apoptosis by direct action on Bcl-2 [49]. Following translocation to the nucleus, JNK activates the transcription factor c-Jun and ATF-2, leading to the formation of Jun-ATF-2 complex activator protein-1 (AP-1). The AP-1 pathway is involved in regulation of pro-apoptotic genes such as TNF-α, Fas-L and Bak [50]. ROS also increase JNK activation through their direct interaction with the upstream mediator of JNK, ASK1 [51]. Under normal conditions, ASK1 remains inactive through binding with thioredoxin (Trx), a ubiquitously expressed protein with a reduction/oxidation active site sequence.

### 3.2. Blood-Brain Barrier (BBB) Disruption

The BBB is a highly selective permeability barrier that separates the circulating blood from the brain extracellular fluid (BECF) in the CNS. Capillary endothelial cells, connected by tight junctions and surrounded by the basal lamina and astrocytic end feet, form the BBB [52]. The BBB allows the selective passage of H_2_O, some gases, and lipid soluble molecules by passive diffusion, and selective transport of molecules such as glucose and amino acids that are crucial to neuronal function whilst protecting the brain by prevention of passage of potential neurotoxins [53].

Following cerebral ischaemia ± reperfusion, generation of ROS (by previously described mechanisms) results in the breakdown of the BBB. Proteases, partly responsible for the breakdown of the BBB by digestion of the basil lamina, are generated within the ischaemic cells of the vasculature and are also released by adhered leukocytes within the lumen [54]. Matrix metalloproteinases (MMPs) expressed in microvascular endothelial cells are a family of proteolytic enzymes, of this family MMP-2 and -9 play key roles in the degradation of the vascular matrix as they digest type IV collagen and laminin, major components of vascular basement membranes [55]. ROS generated within the endothelium during ischaemia cause MMP activation, both directly through oxidation or nitrosylation of MMPs or indirectly through action on the redox-sensitive element of transcription factors (such as NF-κB and AP-1) that are known to be an integral part of the binding sites for MMP transcription [56]. In addition to basal lamina breakdown, expression of the tight and adherent junction proteins responsible for the tight cell-to-cell interactions in the cerebral microvasculature are downregulated following ischaemia by changes in both intra- and extracellular Ca^2+^ ion concentrations, [57,58,59]. Low [Ca^2+^]_i_ results in removal of calcium from binding sites on E-cadherin extracellular domains of adherent junctions, causing a conformational change which interrupts cell-to-cell adhesion inducing BBB breakdown [60]. Within the perivascular space degranulated mast cells release vasoactive mediators such as histamine and proteases, promoting breakdown of the basal lamina from the side of the brain parenchyma. Stress-activated macrophages in the perivascular space release proinflammatory mediators which promote further expression of adhesion molecules on endothelial cells, resulting in more leukocyte adhesion, protease release and subsequent infiltration through the now permeable BBB [61,62,63].

### 3.3. Immune Response

During cerebral ischaemia the inflammatory process is initiated within the occluded vessel. Within the neurovascular unit, the onset of ischaemia results in an initial production of ROS which activates platelets and endothelial cells [64,65]. Within minutes following the onset of ischaemia, pro-inflammatory signals are rapidly generated by translocation of adhesion molecule *p*-selectin to the membrane of the ROS-activated platelets and endothelial cells within the neurovascular unit [66]. Within endothelial cells, the decrease in NO bioavailability (as discussed earlier in this review) results in vasoconstriction, further reducing blood flow to the ischaemic territory and contributing to platelet-leukocyte aggregation. In addition to endothelial derived NO mediated vasoconstriction, oxidative stress within the contractile cells of the capillaries (the pericytes) results in further constriction of the microvasculature [67]. Thrombin generated by ROS-activated platelets causes conversion of fibrinogen to fibrin, the resultant build up of fibrin traps platelets and leukocytes forming clots leading to further microvascular occlusions, and exacerbating the ischaemic insult [68].

In the brain parenchyma microglia, as the innate immune cells of the CNS, are the source of production and release of inflammatory mediators. In the minutes following cerebral ischaemia, there is increased accumulation of ATP or UTP in the extracellular space of the brain parenchyma as a result of excitotoxicity, oedema and subsequent neuronal membrane breakdown [69]. Increased extracellular ATP activates the P2X7 receptors of the microglia, leading to release of pro-inflammatory mediators, such as cytokines, ROS and NO [70].

Under normal conditions, cell-to-cell interaction between neurons and microglia maintains the polarisation and quiescence of the microglia. For example, the membrane protein CD200 expressed on neurons interacts with the CD200R on microglia and enforces the resting phenotype [71]. During ischaemia, expression of this protein is reduced leading to activation of the microglia. In a similar fashion, the cell-surface bound neuronal chemokine, CX3CL1, acts on the microglial CX3CL1 receptor promoting quiescence during normal respiration [72]. Thus, during cerebral ischaemia loss of these interactions by matrix breakdown, releases microglia from suppression and promotes the inflammatory response.

In the later stages following cerebral ischaemia molecular signals are released from the intracellular compartment of dead/dying cells and are produced from the digestion of matrix proteins, known as danger-associated molecular pattern molecules (DAMPs) [73]. DAMPs act on the Toll-like receptors and scavenger receptors of the microglia, perivascular macrophages and brain endothelial cells to stimulate further release of pro-inflammatory mediators such as IL-6 and TNF through NF-κB activation [74]. In addition to this, DAMPs prime dendritic cells for antigen presentation; this interaction demonstrates the main cross over between innate and adaptive immunity following cerebral ischaemia. Although the immune response following stroke is classically thought of in the direction of infiltration from the circulation to the brain parenchyma, it is likely that in the highly vascularised brain the inflammatory mediators released from the brain parenchyma would feed back onto the perivascular unit and the vasculature, reinforcing infiltration of the circulating inflammatory mediators.

## 4. Antioxidant Strategies

In normal brain tissue, ROS are continuously produced during physiological processes but are balanced by endogenous antioxidant defence mechanisms. After cerebral ischaemic injury, free radical production is greatly increased and causes redox disequilibrium in the natural endogenous antioxidant system; detoxification mechanisms are inactivated and oxidants are overproduced. An increase in ROS levels after cerebral ischaemia results in overt oxidative stress and subsequent neuronal injury [75] making free radicals a valid therapeutic target, and much research has focused on assessing the therapeutic effects of antioxidants. There are three main strategies by which antioxidants work; (i) inhibition of free radical production; (ii) scavenging of free radical production; or (iii) increasing free radical degradation [76]. Antioxidant strategies can either focus on the upregulation of endogenous antioxidants or on the delivery of exogenous antioxidants.

### 4.1. Inhibition of Free Radical Production

In this approach, the source of disease promoting ROS generation is targeted by specific inhibitors of ROS generating enzymes. One main source of ROS production in cerebral ischaemia/reperfusion injury is the NADPH oxidases (NOXs). Inhibition of NADPH oxidase complexes with the pharmacological agent apocynin prior to reperfusion has demonstrated an attenuation in cerebral ischaemia in rat models of experimental stroke [77,78] highlighting the contributory role of NOX in brain injury. There are seven homologues of NOX, with NOX2 and NOX4 both shown to be upregulated following I/R injury [79,80]. NOX2 is known to facilitate the production of superoxide, whilst NOX4 produces hydrogen peroxide [81]. NOX2 knockout (KO) mice were found to have decreased lesion volume and improved neurological outcome at 24 h and 72 h post-ischaemic injury with an observed decrease in products of oxidative stress compared to their wild-type (WT) controls [21,82,83]. NOX4 deficient mice have shown protective effects in both transient and permanent models of occlusion at 24 h post-stroke [84]. Furthermore, pharmacological inhibition of TLR4-NOX4 signalling led to reduced expression of NOX4 and a subsequent decrease in volume and area of cerebral infarction of 40% [85]. There are conflicting reports regarding the role of NOX1 in experimental stroke. Studies have shown NOX1 to have a protective role in ischaemic injury in experimental stroke with NOX1 KO mice displaying a four-fold greater cortical infarct volume than WT mice [86]. Similarly, no protection (although no worsening) from experimental stroke was described following tMCAO in NOX1 KO mice in a further study [84]. In contrast, Kahles *et al.* (2010) demonstrated a 55% attenuation in lesion size after 1 h of ischaemia in NOX1 KO mice and a corresponding improvement in neurological improvement compared to WT mice [87]. Interestingly, no difference in lesion volume between WT and NOX1 KO mice was observed when occlusion time was lengthened to 2 h and beyond [87]. Taken together, these data suggest a functional importance for the NOXs in I/R injury and as such represent a novel therapeutic target, especially as apart from their role in ROS production, they have no other essential function [88]. NOX inhibitors are known to be non-specific and not isoform selective; whilst this may not ultimately be important for the treatment of stroke, the development of selective NOX inhibitors would help to validate the role of the various NOX isoforms in stroke [89].

Xanthine Oxidase (XO) is another enzyme that is involved in redox signalling pathways and is an important source of ROS in the setting of brain injury. Inhibition of XO is a potential therapeutic approach for the treatment of cerebral ischaemia that has received little attention. Allopurinol is a commonly used XO inhibitor that not only reduces levels of uric acid, but also reduces the level of superoxide anion formation. Initial trials with this drug are promising; patients treated with allopurinol showed an improvement in vascular [90] and beneficial effects on inflammatory indices compared to placebo [91]. However, in a randomised double-blind trial to investigate the effects of allopurinol in patients with recent subcortical stroke, no improvement in cerebrovascular function was observed [92].

### 4.2. Free Radical Scavengers

Compounds capable of scavenging free radicals have been developed for the treatment of cerebral ischaemic stroke although translation from pre-clinical to clinical trials has largely been disappointing. One of these compounds is Tirilazad mesylate (U-74006F), an inhibitor of lipid peroxidation that was studied extensively in pre-clinical models in the mid-1990s and was shown to reduce infarct size in rats following transient focal ischaemia but not permanent occlusion [93,94]. A meta-analysis of the previously published data was released in 2007 [95], where an overall improvement in both lesion size and neurological recovery was reported. Across 19 publications, tirilazad was demonstrated to reduce lesion size by an average of 29% and improve neurological score by 48% [95]. Maximum efficiency of tirilazad treatment was observed when administered prior to focal ischaemia, with a decreasing efficiency in action with administration time from ischaemic onset thereafter. The largest clinical trial of tirilazad comprised 660 patients, in which tirilazad was administered within 6 h of the onset of cerebral ischaemia [96]. Primary outcome of disability measured by the Glasgow Outcome Scale and Barthel index at 3 months showed no change between groups at an independent interim analysis of 556 patients, and the trial was subsequently terminated. It was later determined that women metabolise tirilazad up to 60% more efficiently than men, and therefore had perhaps not been administered a high enough dose to mediate neuroprotection, reducing the efficacy across the whole trial [97]. These studies highlight the need to more tightly regulate the consistency of methodologies from pre-clinical to clinical studies.

NXY-059 is another example of a drug that showed promising results pre-clinically but failed to show clinical efficacy. A number of pre-clinical studies confirmed the neuroprotective action of the spin trap, NXY-059, in infarct reduction and neurological recovery across a variety of stroke models in both rodents [98,99,100] and non-human primates [101,102]. Spin-trapping is a technique that allows scavenging of free radicals. It involves the addition of a free radical, to a nitrone spin trap resulting in the formation of a spin adduct, without the formation of further free radicals and as such can terminate radical chain reactions. Following extensive and successful pre-clinical studies, NXY-059 was studied in two large randomised and double-blinded trials. The initial trial (SAINT I) involved 1722 patients [103], and the following year SAINT II enrolled 3306 subjects [104]. In both trials, patients were assigned to receive either a 72 h infusion of NXY-059 or placebo, starting within 6 h of the onset of cerebral ischaemia. SAINT I showed a significant improvement in NXY-059 treated patients assessed by the modified Rankin score, but not by the NIHSS scale or Barthel index. However, the subsequent SAINT II trial published entirely negative results in primary and secondary endpoints. The difference in the outcomes of these trials have been attributed to statistical weakness of the SAINT I trial [105,106] and the poor BBB permeability of NXY-059 [107].

Edaravone is a free radical scavenger that has been approved for use in Japan since 2001 [108] and is widely in use in clinics in Japan for the treatment of cerebral infarctions [109]. It is known to scavenge peroxyl, hydroxyl and superoxide radicals [110]. Although free radicals are known to be a major contributor to lesion progression, the effectiveness of edaravone is still unclear. Pre-clinical studies show promising results with decreased infarct sizes in rodent models [111,112,113,114]. In human clinical trials, the results are less clear however. In a multicentre, randomized, placebo-controlled double-blind study on acute ischaemic stroke patients, significant clinical improvements in all patients receiving edaravone and at all time points were described [115]. Conversely, in patients with cardioembolic stroke treatment with edaravone showed minimal improvements [116]. In a further study to assess the effects of edaravone in both acute and chronic stages of cerebral ischaemia, infarct size was significantly reduced in small-vessel occlusion stroke patients within one year; however associated observed neurological improvements were not significantly sustained at one year [117]. Drug dose and therapeutic window for treatment is not consistent between all these trials and so a trial that addresses these issues is warranted to fully assess the efficacy profile of edaravone in stroke patients [109].

### 4.3. Free Radical Degradation

Strategies aimed at reducing oxidative stress by increasing levels of the antioxidant SOD in experimental models of stroke have demonstrated the integral role of ROS in lesion progression. SOD catalyses the conversion of O_2_^−^ to less reactive H_2_O_2_ and O_2_. Catalase (CAT) and glutathione peroxidase (GPx) help to eliminate the by-product of H_2_O_2_, thus increasing the overall efficacy of SOD [118]. Overexpression of CAT by adenoviral vectors [119] and transduction with PEP-1-CAT fusion protein [120] have both demonstrated neuroprotection in neuronal cells *in vitro* against I/R injury. Furthermore, catalase overexpression was shown to be protective when delivered prior to ischaemic injury in rats; however protection was lost when delivery was after the ischaemic insult [121]. Transgenic mice overexpressing GPx showed significant reduction in infarct volume compared to non-transgenic mice following I/R damage [122,123] whilst Gpx-1 knockout (KO) mice demonstrated a three-fold increase in infarct volume [124].

Of the three SOD enzyme isoforms, SOD1 has been studied most in relation to experimental models of stroke. Approaches to overexpress SOD1 using transgenic mice [125] and rats [37] demonstrated reduced apoptosis in transient focal ischaemia stroke models. Conversely, deficient SOD1 expression in knockout mice resulted in mortality within 24 h of MCAO in SOD1^−/−^ mice and increased infarct volume and oedema in SOD1^+/−^ heterozygotes compared to WT controls [126]. Using gene therapy to overexpress SOD1 locally before and 2 h post-transient MCAO a significant improvement in neuronal survival was seen [127]. Furthermore, transient MCAO in transgenic mice overexpressing SOD3 resulted in reduced infarct size compared to wild type mice [128]. SOD2 (or manganese-containing superoxide dismutase, Mn-SOD) is a mitochondrial antioxidant enzyme. Whilst homozygous SOD2 KO mice (SOD2^−/−^) die within 10 days of birth [129], heterozygotes (SOD2^+/−^) have increased levels of superoxide and show an increase in infarct volume after cerebral ischaemia compared to WT [130] suggesting that SOD2 protects against oxidative stress-induced damage. Transgenic mice overexpressing SOD2 displayed neuroprotective effects following transient focal ischaemia [131]. SOD2 was revealed to be a gene that is a specific target of STAT3 with the loss of STAT3 activity by ischemic reperfusion resulting in a reduction of SOD2 expression [132]. Injections of interleukin-6 (IL-6) before and after middle cerebral artery occlusion in mice restored activity of STAT3 and additionally restored the transcriptional activity of the Mn-SOD promoter through recovery of the recruitment of STAT3 to the Mn-SOD promoter; a resulting reduction in infarct volume was observed [133]. Thus targeting these pathways may have therapeutic potential against oxidative stress in cerebral infarction.

Ebselen is an inhibitor of glutathione peroxidase-like activity, and also reacts with and subsequently scavenges ONOO^−^. In pre-clinical rodent models of focal ischaemia pre-treatment with ebselen [134] or administration at point of reperfusion [135] in transient occlusion models improved ischaemic damage and neurological deficit, respectively. Post-treatment at 30 min following onset of ischaemia in a rodent model of permanent occlusion resulted in modest protection [136]. However, a randomised and blinded trial of 302 ischaemic stroke patients who were administered ebselen at 48 h post ischaemia for 2 weeks failed to replicate the protective effects seen in the pre-clinical models at 3 months, although improvements in the ebselen treated groups were observed prior to this at 1 month [137].

A novel antioxidant approach is the inhalation of gases during or after ischaemia. The use of inhaled hydrogen gas to selectively reduce the hydroxyl radical in a transient model of MCAO demonstrated a marked reduction in infarct volume 1 day post-occlusion and an improvement in neurological scores assessed after 7 days [138]. Importantly, gas administration during reperfusion was adequate to elicit the beneficial effect. Normobaric oxygen (NBO) has been shown in several studies to reduce infarct volume and neurological deficits in rat models of focal ischaemia [139,140,141] and to extend the time window of successful reperfusion in rats [142]. Furthermore, combination therapy with NBO and ethanol was shown to have neuroprotective effects after I/R injury in rats [143,144]. A similar approach to the treatment of ischaemic injury is the induction of a natural cell protection molecule NO. NO is a vasoactive molecule that is produced by either endothelial NO synthase (eNOS), inducible NO synthase (iNOS) or neuronal NO synthase (nNOS) with NO playing a dual contradictory role in focal cerebral ischaemia [145,146]. NO derived from eNOS has neuroprotective effects [67] and can terminate chain reactions during lipid peroxidation, whilst NO derived from iNOS acts as a pro-oxidant and reacts with superoxide O_2_^−^ to form the strongly oxidative/nitrative molecule ONOO^−^ and ultimately exacerbates cell death [147]. In a recent study, it was shown that inhalation of NO could significantly decrease ischaemic brain damage and improve neurological outcome in rat neonatal stroke models [148], murine models of cerebral ischaemia and a large animal model of ischaemic stroke [149]. Gaseous treatment may have several therapeutic advantages including the ability of gases to rapidly penetrate biomembranes and diffuse into the cytosol, mitochondria and nucleus [138].

Lubeluzole acts to reduce NO levels and subsequent ONOO^−^ production in hypoxic cells through inhibition of the glutamate-mediated nitric oxide synthase pathway [150]. Proof-of-concept was confirmed *in vitro*, lubeluzole protected both hippocampal slices [151] and primary neurons [152] from both membrane depolarisations and nitric oxide toxicity, respectively. In addition, in a pre-clinical model of photochemical parietal sensorimotor cortical stroke in rats, lubeluzole rescued hindlimb placing when administered 5 min following ischaemic onset in all rats and 60% of rats when administered at 6 h [153]. In a transient middle cerebral artery occlusion (MCAO) model lubeluzole treatment 15 minutes after the onset of stroke rescued infarct by 50% [154]. When treatment of lubeluzole was administered 3 h following the onset of permanent MCA, infarct was reduced by 33% [154]. Primary clinical safety studies of lubeluzole in 193 patients were terminated early as a result of a mortality imbalance in the higher dose group (20 mg/day) which was not noted in the lower dose group (10 mg/day) [155]. The following year a multicentre randomised and double-blinded trial of 721 patients was carried out in the US and Canada, where patients were randomised to lubeluzole or placebo administration within 6 h following ischaemic onset. Mortality was not improved at 12 weeks, improvement was demonstrated in the lubeluzole groups across the NIHSS scale and Barthel index at the same timepoint [156]. These results were confirmed in a similar study of comparable candidate number the following year [157]. In light of these positive trials, a large clinical study of 1786 patients was initiated, however unfortunately no difference was observed between lubeluzole or placebo groups in either the primary or secondary outcome measures [158]. A meta-analysis of all five clinical trials of lubeluzole reported no improvement of mortality or dependency between groups, but did report a significant increase in the heart-conduction disorder, Q-T prolongation, in lubeluzole treated subjects [159].

### 4.4. Mitochondrial Targeted Anti-Oxidants

The mitochondrial matrix is an important site of free radical generation [160]. Mitochondria have been reported to act as major sources of ROS in ischaemia and particularly in reperfusion injury. Oxidative damage to the mitochondria can result in a decrease in essential ATP production, an increase in mitochondrial generated ROS production and in the release of pro-apoptotic signals. The MRC, which is made up of four membrane-bound complexes (I-IV), has been identified as one potential source of ROS production [161]. Inhibition of the mitochondrial complex I was found to inhibit both ischaemic and reperfusion-mediated oxidative damage protecting the mouse brain from hypoxic/ischaemic (HI) injury [19].

A lack of efficacy in the use of antioxidants can in part be explained by a difficulty in achieving high concentrations in the necessary intracellular location [162]. Mitochondrial targeted antioxidants could specifically target the interior of the mitochondrion and potentially ameliorate oxidative damage. Targeting antioxidants to the mitochondria generally involves conjugating an antioxidant to a lipophilic cation to promote diffusion and accumulation within the mitochondria. A number of antioxidants have been targeted to the mitochondria in an attempt to improve efficacy. Mitochondrial targeted vitamin E was shown to protect cerebellar granule cells *in vitro* from ethanol-induced oxidative damage [163]. Additionally, supplementation of bovine aortic endothelial cells with mitochondrial targeted vitamin E mitigates peroxide-mediated oxidative stress and inhibits apotosis [164].

Mitoquinone (mitoQ) is a derivative of ubiquinone and has a high affinity for the mitochondria [165]. MitoQ_10_ is reduced to ubiquinol and has been found to be an effective antioxidant protecting mitochondria from oxidative damage and apoptosis caused by H_2_O_2_ [166]. It has been shown to be therapeutic in several animal models of disease and in humans (reviewed in [167]). Pretreatment of rats for 2 weeks prior to *ex vivo* cardiac ischaemia/reperfusion demonstrated cardiac protection [168]. Furthermore, administration of MitoQ_10_ prevented hypertension, cardiac hypertrophy and improved endothelial function after oral administration in young spontaneously hypertensive stroke prone rats (SHRSP) [169,170]. Administration of MitoQ_10_ reduced levels of oxidative stress and cell death in rat brain induced by chemical treatment with the organophosphate pesticide dichlorvos [171]. However, in a rat model of neonatal HI no such protection was described for MitoQ [172]. Thus mitochondrial targeted antioxidants represent an important developing therapeutic strategy in the treatment of stroke.

### 4.5. Upregulation of Endogenous Antioxidants

Antioxidant vitamins are one of the body’s main natural defence mechanisms against oxidative stress. Vitamin E and C are two of the most studied natural antioxidants. Dietary vitamin C is mostly provided through the consumption of fruit and vegetables and has a biological role as a hydrogen donor to reverse oxidation. One form of vitamin C, ascorbic acid, was found to protect newborn rat brain from HI injury [173]. In a large animal stroke model, the administration of dehydroascorbic acid did not significantly decrease infarct volume or improve neurological outcome and the study was terminated early [174]. In a recent study however, it was found that 4 weeks of pre-treatment of stroke-prone spontaneously hypertensive rats with vitamins C and E lowered levels of lipid peroxidation and significantly lowered infarct volume following MCAO [175]. In large human observational studies, it was determined that an increase in vitamin C plasma levels correlated with a lowered incidence of stroke [176,177,178]. However, in randomised control trials, patients receiving antioxidant vitamin supplementation displayed no difference in incidence of stroke compared to those receiving placebo [179,180,181]. Similar results were found for vitamin E supplementation with meta-analysis of randomised control trials showing no benefit on the incidence of stroke in patients receiving supplementation compared to those taking placebo [182,183]. These data would suggest that the use of antioxidant vitamin supplementation in stroke is not therapeutically viable.

Hypoxia-inducible factor 1 (HIF-1) is an important mediator in stroke and is responsible for the inductions of genes that are involved in the cell’s survival response to hypoxia [184]. The neuroprotective effects of HIF-1 have been well documented in pre-clinical models. Pre-conditioning of the brain 24 h prior to ischaemic insult has been shown to reduce infarct volume by up to 30% [185] through the increase in expression of HIF-1 and its target genes [186]. Additionally, pre-treatment of rats with deferoxamine, a known inducer of HIF-1, demonstrated significant protection against ischaemic injury [187,188]. This effect may also be attributed to the ability of deferoxamine to chelate Fe^2+^ and inhibit the formation of the damaging •OH radical through the Fenton reaction.

The exact function and mechanism of neuroglobin (Ngb), a member of the globin superfamily, remains elusive but its upregulation has been shown to be neuroprotective in a number of studies of *in vivo* brain ischaemia models. Transgenic mice overexpressing Ngb showed neuroprotective effects in the form of reduced infarct volume and reduced lipid peroxidation levels following both focal [189] and global ischaemia [190]. Adeno-associated viral mediated Ngb expression also reduced infarct size and improved neurological outcome 24 h post-stroke in rats [191]. The use of cell-penetrating peptides to deliver Ngb across the blood brain barrier in mice resulted in significantly reduced lesion size and improved neurological recovery when given as a pre-treament [192]. No improvement was seen when delivered post stroke, however. In a recent combination study to assess the effects of targeting both oxidative stress and apoptotic pathways using a combination of the antiapoptotic c-Jun *N*-terminal kinase (JNK) inhibitor and the antioxidant neuroglobin (Ngb), combined treatment reduced infarct and improved neurological outcome more than single therapy after *in vivo* experimental stroke in hypertensive rats [193]. This demonstrates how targeting multiple physiological pathways can be beneficial.

### 4.6. Combination Therapy

No single antioxidant has advanced beyond clinical trials in the UK and the only approved treatment remains tissue-plasminogen activator (tPA). Few patients receive this treatment, as a result of its narrow treatment window of 4.5 h post-stroke [194], making stroke a vastly undertreated disease. A promising strategy in the treatment of stroke is the identification of agents that when used in combination may have increased efficacy in stroke treatment compared to single-agent therapy. This approach has proven successful in other pathologies of CVD. As many neuroprotective trials have failed due to dose-limiting toxicity, combination therapy may decrease the dose required for each agent so reducing adverse events. It has been shown that combination with activated protein C reduces tPA associated neurovascular toxicity thus improving efficacy in the treatment of stroke [195]. Although complicating study design, multimodal therapy would allow the targeting of multiple pathophysiological mechanisms. Indeed, our own studies in stroke combining stem cell therapy and targeting oxidative stress and matrix metalloproteinases found that triple therapy was more effective than single or double combinations [196]. Furthermore, our previously discussed study combining the antioxidant Ngb with the antiapoptotic JNK inhibitor demonstrated improved outcome following ischaemia with combination therapy compared to either single therapy alone [193].

Indeed, the majority of pre-clinical studies indicate that treatment with thrombolytics is suboptimal if not in combination with neuroprotective agents. At least additive but also synergistic effects have been demonstrated by combination of thrombolytics with neuroprotectants in pre-clinical models including the free radical spin trap α-Phenyl-*tert*-butyl-nitrone (PBN) [197]. In this experiment, tPA-induced haemorrhage volumes were reduced by 40% with alpha-PBN, and infarction and neurological deficits were also decreased. PBN has also been used in combination with NMDA receptor antagonist (MK-801) in an *in vitro* model of OGD and demonstrated substantial synergistic effects in combination [198]. The use of tPA in combination with the free radical scavenger edaravone prevented the reduction in levels of protein factors relating to neurorepair and neuroregeneration and decreased infarct volume when both were delivered during stroke surgery in rats [199]. However, disappointingly, NBO administration in combination with tPA showed no additional beneficial neuroprotective effects in a rat model of thromboembolic stroke [200]. The volume of ischaemic brain damage and swelling in these animals was equivalent to that of control animals and greater than that of tPA- and NBO- treated animals suggesting this may not be a safe strategy. Synergistic effects have been observed with two different antioxidants, U-74389G and U-101033E. Sprague-Dawley rats were subjected to 90 min tMCAO, and treatment was administered 15 min prior to ischaemia, during ischaemia 15 min prior to reperfusion and 45 min following reperfusion. Synergistic improvement was observed in functional recovery but no improvement with combined therapy was noted regarding lesion size at 7 days [201].

A meta-analysis of combination therapy used in stroke research found that of the 126 treatments tested, single treatments reduced infarct size by 20% and improved neurological score by 12% compared with control; whilst a second therapy improved efficacy by an additional 18% and 25%, respectively [202]. When used in combination with thrombolytics, combined therapies can increase the therapeutic time window up to 8.8 h in animal models [202]. Combinations of neuroprotective agents thus represent a relatively new field of stroke therapy, with incredibly large potential.

### 4.7. Reasons for Failure?

Despite advancement in the understanding of the pathophysiology of stroke and a vast effort in therapeutic research, many clinical trials have failed regardless of their success at the pre-clinical stage [203]. The reason behind this is unclear but a number of factors may play a part in this anomaly, for example factors such as, the animal model, physiological monitoring and outcome measures; the majority of pre-clinical experiments are carried out on young, male animals without comorbidities [202]. For this reason, and in order to address these issues, a conference of academics and industry representatives was convened to suggest a set of guidelines for the evaluation of pre-clinical therapies known as the Stroke Therapy Academic Industry Roundtable (STAIR) initially in 1999 [204], and reviewed in 2009 [205]. A systematic review by O’Collins *et al.* in 2006 [206] of ~3500 articles published addressing various neuroprotective strategies between 1957 and 2003 showed that only five out of 550 of the drugs reported to be effective fully met the standards set by the STAIR guidelines [206]. One of the main findings within the review was a lack of randomisation and blinding, resulting in an overestimated efficacy of therapies. In fact, although there has been an overall trend towards improvement in stroke study design, only 36% of studies report randomisation, 11% report concealment, 29% report blinded assessment of outcome, and 3% reported use of power calculations in generation of sample size [207]. To increase the chance of successful translation of pre-clinical to clinical, it is essential to improve study design and to test across a range of models as no single model is likely to represent the heterogenic nature of stroke [208].

## 5. Conclusions

While there is no disputing the deleterious effects and detrimental contribution of ROS to lesion progression following ischaemic stroke there remains doubt over the clinical efficacy of anti-oxidants in this setting. Many strategies have proved therapeutic in the pre-clinical setting with translation to the clinic failing to replicate benefit. Efforts to improve the validity of existing animal models through the revised STAIR [205], pre-clinical stroke [209] and ARRIVE [210] guidelines along with the potential implementation of international multicentre pre-clinical stroke studies [211] will, undoubtedly, result in improved clinical translation for stroke therapeutics. However, many potentially beneficial antioxidant strategies with proven efficacy from robust animal studies remain worthy of clinical investigation. Advances in knowledge of the source and nature of these ROS will lead to further new directions to interrogate. Finally, the potential to combine anti-oxidant protection with existing thrombolytics and novel neuroprotectants has yet to be fully established and may represent a powerful means to improve outcome after this devastating and debilitating ischaemic event.

## References

[1] Lozano R., Naghavi M., Foreman K., Lim S., Shibuya K., Aboyans V., Abraham J., Adair T., Aggarwal R., Ahn S.Y. (2012). Global and regional mortality from 235 causes of death for 20 age groups in 1990 and 2010: A systematic analysis for the global burden of disease study 2010. Lancet.

[2] Murray C.J.L., Vos T., Lozano R., Naghavi M., Flaxman A.D., Michaud C., Ezzati M., Shibuya K., Salomon J.A., Abdalla S. (2012). Disability-adjusted life years (dalys) for 291 diseases and injuries in 21 regions, 1990–2010: A systematic analysis for the global burden of disease study 2010. Lancet.

[3] Townsend N., Wickramasinghe K., Bhatnagar P., Smolina K., Nichols M., Leal J., Luengo-Fernandez R., Rayner M. (2012). Coronary Heart Disease Statistics.

[4] Li B.-H., Ding X., Yin Y.-W., Liu Y., Gao C.-Y., Zhang L.-L., Li J.-C. (2013). Meta-analysis of clinical outcomes of intravenous recombinant tissue plasminogen activator for acute ischemic stroke: Within 3 h *versus* 3–4.5 h. Curr. Med. Res. Opin..

[5] Adeoye O., Hornung R., Khatri P., Kleindorfer D. (2011). Recombinant tissue-type plasminogen activator use for ischemic stroke in the united states: A doubling of treatment rates over the course of 5 years. Stroke.

[6] Herculano-Houzel S. (2011). Scaling of brain metabolism with a fixed energy budget per neuron: Implications for neuronal activity, plasticity and evolution. PLoS One.

[7] Pauwels P.J., Opperdoes F.R., Trouet A. (1985). Effects of antimycin, glucose deprivation, and serum on cultures of neurons, astrocytes, and neuroblastoma cells. J. Neurochem..

[8] Uttara B., Singh A.V., Zamboni P., Mahajan R.T. (2009). Oxidative stress and neurodegenerative diseases: A review of upstream and downstream antioxidant therapeutic options. Curr. Neuropharmacol..

[9] Kahles T., Brandes R.P. (2012). NADPH oxidases as therapeutic targets in ischemic stroke. Cell. Mol. Life Sci..

[10] Sanderson T.H., Reynolds C.A., Kumar R., Przyklenk K., Huttemann M. (2013). Molecular mechanisms of ischemia-reperfusion injury in brain: Pivotal role of the mitochondrial membrane potential in reactive oxygen species generation. Mol. Neurobiol..

[11] Vergeade A., Mulder P., Vendeville C., Ventura-Clapier R., Thuillez C., Monteil C. (2012). Xanthine oxidase contributes to mitochondrial ros generation in an experimental model of cocaine-induced diastolic dysfunction. J. Cardiovasc. Pharmacol..

[12] Rice M.E. (2011). H2O2: A dynamic neuromodulator. Neuroscientist.

[13] Liu S., Shi H., Liu W., Furuichi T., Timmins G.S., Liu K.J. (2004). Interstitial pO2 in ischemic penumbra and core are differentially affected following transient focal cerebral ischemia in rats. J. Cereb. Blood Flow Metab..

[14] Ying W., Han S.K., Miller J.W., Swanson R.A. (1999). Acidosis potentiates oxidative neuronal death by multiple mechanisms. J. Neurochem..

[15] Stanika R.I., Villanueva I., Kazanina G., Andrews S.B., Pivovarova N.B. (2012). Comparative impact of voltage-gated calcium channels and nmda receptors on mitochondria-mediated neuronal injury. J. Neurosci..

[16] Yamato M., Egashira T., Utsumi H. (2003). Application of *in vivo* ESR spectroscopy to measurement of cerebrovascular ROS generation in stroke. Free Radic. Biol. Med..

[17] Peters O., Back T., Lindauer U., Busch C., Megow D., Dreier J., Dirnagl U. (1998). Increased formation of reactive oxygen species after permanent and reversible middle cerebral artery occlusion in the rat. J. Cereb. Blood Flow Metab..

[18] Chen Q., Moghaddas S., Hoppel C.L., Lesnefsky E.J. (2008). Ischemic defects in the electron transport chain increase the production of reactive oxygen species from isolated rat heart mitochondria. Am. J. Physiol. Cell. Physiol..

[19] Niatsetskaya Z.V., Sosunov S.A., Matsiukevich D., Utkina-Sosunova I.V., Ratner V.I., Starkov A.A., Ten V.S. (2012). The oxygen free radicals originating from mitochondrial complex I contribute to oxidative brain injury following hypoxia-ischemia in neonatal mice. J. Neurosci..

[20] Chouchani E.T., Methner C., Nadtochiy S.M., Logan A., Pell V.R., Ding S., James A.M., Cocheme H.M., Reinhold J., Lilley K.S. (2013). Cardioprotection by *S*-nitrosation of a cysteine switch on mitochondrial complex I. Nat. Med..

[21] Chen H., Song Y.S., Chan P.H. (2009). Inhibition of NADPH oxidase is neuroprotective after ischemia-reperfusion. J. Cereb. Blood Flow Metab..

[22] Bokoch G.M., Knaus U.G. (2003). NADPH oxidases: Not just for leukocytes anymore!. Trends Biochem. Sci..

[23] Cheng G., Ritsick D., Lambeth J.D. (2004). Nox3 regulation by NOXO1, p47phox, and p67phox. J. Biol. Chem..

[24] Matsushima S., Kuroda J., Ago T., Zhai P., Park J.Y., Xie L.H., Tian B., Sadoshima J. (2013). Increased oxidative stress in the nucleus caused by Nox4 mediates oxidation of HDAC4 and cardiac hypertrophy. Circ. Res..

[25] Granger D.N., Rutili G., McCord J.M. (1981). Superoxide radicals in feline intestinal ischemia. Gastroenterology.

[26] Parks D.A., Granger D.N. (1986). Xanthine oxidase: Biochemistry, distribution and physiology. Acta Physiol. Scand. Suppl..

[27] Crack P.J., Taylor J.M. (2005). Reactive oxygen species and the modulation of stroke. Free Radic. Biol. Med..

[28] Gursoy-Ozdemir Y., Can A., Dalkara T. (2004). Reperfusion-induced oxidative/nitrative injury to neurovascular unit after focal cerebral ischemia. Stroke.

[29] Nelson C.W., Wei E.P., Povlishock J.T., Kontos H.A., Moskowitz M.A. (1992). Oxygen radicals in cerebral ischemia. Am. J. Physiol..

[30] McCracken E., Valeriani V., Simpson C., Jover T., McCulloch J., Dewar D. (2000). The lipid peroxidation by-product 4-hydroxynonenal is toxic to axons and oligodendrocytes. J. Cereb. Blood Flow Metab..

[31] Matsuda S., Umeda M., Uchida H., Kato H., Araki T. (2009). Alterations of oxidative stress markers and apoptosis markers in the striatum after transient focal cerebral ischemia in rats. J. Neural Transm..

[32] Liu P.K., Hsu C.Y., Dizdaroglu M., Floyd R.A., Kow Y.W., Karakaya A., Rabow L.E., Cui J.K. (1996). Damage, repair, and mutagenesis in nuclear genes after mouse forebrain ischemia-reperfusion. J. Neurosci..

[33] Chen J., Jin K., Chen M., Pei W., Kawaguchi K., Greenberg D.A., Simon R.P. (1997). Early detection of DNA strand breaks in the brain after transient focal ischemia: Implications for the role of DNA damage in apoptosis and neuronal cell death. J. Neurochem..

[34] Nagayama T., Simon R.P., Chen D., Henshall D.C., Pei W., Stetler R.A., Chen J. (2000). Activation of poly(adp-ribose) polymerase in the rat hippocampus may contribute to cellular recovery following sublethal transient global ischemia. J. Neurochem..

[35] Kawase M., Fujimura M., Morita-Fujimura Y., Chan P.H. (1999). Reduction of apurinic/apyrimidinic endonuclease expression after transient global cerebral ischemia in rats: Implication of the failure of DNA repair in neuronal apoptosis. Stroke.

[36] Kirkland R.A., Windelborn J.A., Kasprzak J.M., Franklin J.L. (2002). A bax-induced pro-oxidant state is critical for cytochrome c release during programmed neuronal death. J. Neurosci..

[37] Sugawara T., Lewen A., Gasche Y., Yu F., Chan P.H. (2002). Overexpression of SOD1 protects vulnerable motor neurons after spinal cord injury by attenuating mitochondrial cytochrome c release. FASEB J..

[38] Croall D.E., DeMartino G.N. (1991). Calcium-activated neutral protease (calpain) system: Structure, function, and regulation. Physiol. Rev..

[39] Scorrano L., Penzo D., Petronilli V., Pagano F., Bernardi P. (2001). Arachidonic acid causes cell death through the mitochondrial permeability transition. Implications for tumor necrosis factor-alpha aopototic signaling. J. Biol. Chem..

[40] Enari M., Sakahira H., Yokoyama H., Okawa K., Iwamatsu A., Nagata S. (1998). A caspase-activated dnase that degrades DNA during apoptosis, and its inhibitor icad. Nature.

[41] Jänicke R.U., Sprengart M.L., Wati M.R., Porter A.G. (1998). Caspase-3 is required for DNA fragmentation and morphological changes associated with apoptosis. J. Biol. Chem..

[42] Hanus J., Kalinowska-Herok M., Widlak P. (2008). The major apoptotic endonuclease DFF40/CAD is a deoxyribose-specific and double-strand-specific enzyme. Apoptosis.

[43] Widlak P., Li P., Wang X., Garrard W.T. (2000). Cleavage preferences of the apoptotic endonuclease DFF40 (caspase-activated dnase or nuclease) on naked DNA and chromatin substrates. J. Biol. Chem..

[44] Dalton T.P., Shertzer H.G., Puga A. (1999). Regulation of gene expression by reactive oxygen. Annu. Rev. Pharmacol. Toxicol..

[45] Pautz A., Art J., Hahn S., Nowag S., Voss C., Kleinert H. (2010). Regulation of the expression of inducible nitric oxide synthase. Nitric Oxide.

[46] Kim E.J., Raval A.P., Hirsch N., Perez-Pinzon M.A. (2010). Ischemic preconditioning mediates cyclooxygenase-2 expression via nuclear factor-kappa B activation in mixed cortical neuronal cultures. Transl. Stroke Res..

[47] Hsieh H.L., Wang H.H., Wu W.B., Chu P.J., Yang C.M. (2010). Transforming growth factor-beta1 induces matrix metalloproteinase-9 and cell migration in astrocytes: Roles of ros-dependent ERK- and JNK-NF-kappaB pathways. J. Neuroinflamm..

[48] Park T.Y., Baik E.J., Lee S.H. (2013). Prostaglandin e(2)-induced intercellular adhesion molecule-1 expression is mediated by cAMP/Epac signalling modules in bEnd.3 brain endothelial cells. Br. J. Pharmacol..

[49] Deng X., Xiao L., Lang W., Gao F., Ruvolo P., May W.S. (2001). Novel role for JNK as a stress-activated Bcl2 kinase. J. Biol. Chem..

[50] Fan M., Chambers T.C. (2001). Role of mitogen-activated protein kinases in the response of tumor cells to chemotherapy. Drug Resist. Updat.

[51] Soberanes S., Urich D., Baker C.M., Burgess Z., Chiarella S.E., Bell E.L., Ghio A.J., de Vizcaya-Ruiz A., Liu J., Ridge K.M. (2009). Mitochondrial complex III-generated oxidants activate ASK1 and JNK to induce alveolar epithelial cell death following exposure to particulate matter air pollution. J. Biol. Chem..

[52] De Vries H.E., Kuiper J., de Boer A.G., van Berkel T.J., Breimer D.D. (1997). The blood-brain barrier in neuroinflammatory diseases. Pharmacol. Rev..

[53] Grieb P., Forster R.E., Strome D., Goodwin C.W., Pape P.C. (1985). O_2_ exchange between blood and brain tissues studied with ^18^O_2_ indicator-dilution technique. J. Appl. Physiol..

[54] Neumann-Haefelin T., Kastrup A., de Crespigny A., Yenari M.A., Ringer T., Sun G.H., Moseley M.E. (2000). Serial mri after transient focal cerebral ischemia in rats: Dynamics of tissue injury, blood-brain barrier damage, and edema formation. Stroke.

[55] Rosell A., Cuadrado E., Ortega-Aznar A., Hernández-Guillamon M., Lo E.H., Montaner J. (2008). Mmp-9-positive neutrophil infiltration is associated to blood-brain barrier breakdown and basal lamina type IV collagen degradation during hemorrhagic transformation after human ischemic stroke. Stroke.

[56] Cortez D.M., Feldman M.D., Mummidi S., Valente A.J., Steffensen B., Vincenti M., Barnes J.L., Chandrasekar B. (2007). Il-17 stimulates MMP-1 expression in primary human cardiac fibroblasts via p38 MAPK- and ERK1/2-dependent C/EBP-β, NF-κB, and AP-1 activation. Am. J. Physiol. Heart Circ. Physiol..

[57] Mark K.S., Davis T.P. (2002). Cerebral microvascular changes in permeability and tight junctions induced by hypoxia-reoxygenation. Am. J. Physiol. Heart Circ. Physiol..

[58] Huber J.D., Witt K.A., Hom S., Egleton R.D., Mark K.S., Davis T.P. (2001). Inflammatory pain alters blood-brain barrier permeability and tight junctional protein expression. Am. J. Physiol. Heart Circ. Physiol..

[59] Yamagata K., Tagami M., Takenaga F., Yamori Y., Itoh S. (2004). Hypoxia-induced changes in tight junction permeability of brain capillary endothelial cells are associated with IL-1beta and nitric oxide. Neurobiol. Dis..

[60] Pokutta S., Herrenknecht K., Kemler R., Engel J. (1994). Conformational changes of the recombinant extracellular domain of E-cadherin upon calcium binding. Eur. J. Biochem..

[61] Cipolla M.J., Crete R., Vitullo L., Rix R.D. (2004). Transcellular transport as a mechanism of blood-brain barrier disruption during stroke. Front. Biosci..

[62] Xu H., Dawson R., Crane I.J., Liversidge J. (2005). Leukocyte diapedesis *in vivo* induces transient loss of tight junction protein at the blood-retina barrier. Investig. Ophthalmol. Vis. Sci..

[63] Konsman J.P., Drukarch B., van Dam A.M. (2007). (Peri)vascular production and action of pro-inflammatory cytokines in brain pathology. Clin. Sci..

[64] Peerschke E.I., Yin W., Ghebrehiwet B. (2010). Complement activation on platelets: Implications for vascular inflammation and thrombosis. Mol. Immunol..

[65] Pinsky D.J., Naka Y., Liao H., Oz M.C., Wagner D.D., Mayadas T.N., Johnson R.C., Hynes R.O., Heath M., Lawson C.A. (1996). Hypoxia-induced exocytosis of endothelial cell weibel-palade bodies. A mechanism for rapid neutrophil recruitment after cardiac preservation. J. Clin. Investig..

[66] Yilmaz G., Granger D.N. (2010). Leukocyte recruitment and ischemic brain injury. Neuromol. Med..

[67] Atochin D.N., Wang A., Liu V.W., Critchlow J.D., Dantas A.P., Looft-Wilson R., Murata T., Salomone S., Shin H.K., Ayata C. (2007). The phosphorylation state of enos modulates vascular reactivity and outcome of cerebral ischemia *in vivo*. J. Clin. Investig..

[68] Del Zoppo G.J., Schmid-Schonbein G.W., Mori E., Copeland B.R., Chang C.M. (1991). Polymorphonuclear leukocytes occlude capillaries following middle cerebral artery occlusion and reperfusion in baboons. Stroke.

[69] Melani A., Turchi D., Vannucchi M.G., Cipriani S., Gianfriddo M., Pedata F. (2005). ATP extracellular concentrations are increased in the rat striatum during *in vivo* ischemia. Neurochem. Int..

[70] Korcok J., Raimundo L.N., Ke H.Z., Sims S.M., Dixon S.J. (2004). Extracellular nucleotides act through P2X7 receptors to activate NF-kappaB in osteoclasts. J. Bone Miner. Res..

[71] Lyons A., Downer E.J., Crotty S., Nolan Y.M., Mills K.H., Lynch M.A. (2007). CD200 ligand receptor interaction modulates microglial activation *in vivo* and *in vitro*: A role for IL-4. J. Neurosci..

[72] Denes A., Ferenczi S., Halasz J., Kornyei Z., Kovacs K.J. (2008). Role of CX3CR1 (fractalkine receptor) in brain damage and inflammation induced by focal cerebral ischemia in mouse. J. Cereb. Blood Flow Metab..

[73] Chen G.Y., Nunez G. (2010). Sterile inflammation: Sensing and reacting to damage. Nat. Rev. Immunol..

[74] Marsh B.J., Williams-Karnesky R.L., Stenzel-Poore M.P. (2009). Toll-like receptor signaling in endogenous neuroprotection and stroke. Neuroscience.

[75] Facchinetti F., Dawson V.L., Dawson T.M. (1998). Free radicals as mediators of neuronal injury. Cell. Mol. Neurobiol..

[76] Margaill I., Plotkine M., Lerouet D. (2005). Antioxidant strategies in the treatment of stroke. Free Radic. Biol. Med..

[77] Tang L.L., Ye K., Yang X.F., Zheng J.S. (2007). Apocynin attenuates cerebral infarction after transient focal ischaemia in rats. J. Int. Med. Res..

[78] Genovese T., Mazzon E., Paterniti I., Esposito E., Bramanti P., Cuzzocrea S. (2011). Modulation of NADPH oxidase activation in cerebral ischemia/reperfusion injury in rats. Brain Res..

[79] McCann S.K., Dusting G.J., Roulston C.L. (2008). Early increase of NOX4 NADPH oxidase and superoxide generation following endothelin-1-induced stroke in conscious rats. J. Neurosci. Res..

[80] Yoshioka H., Niizuma K., Katsu M., Okami N., Sakata H., Kim G.S., Narasimhan P., Chan P.H. (2011). NADPH oxidase mediates striatal neuronal injury after transient global cerebral ischemia. J. Cereb. Blood Flow Metab..

[81] Serrander L., Cartier L., Bedard K., Banfi B., Lardy B., Plastre O., Sienkiewicz A., Forro L., Schlegel W., Krause K.H. (2007). NOX4 activity is determined by MRNA levels and reveals a unique pattern of ros generation. Biochem. J..

[82] Chen H., Kim G.S., Okami N., Narasimhan P., Chan P.H. (2011). NADPH oxidase is involved in post-ischemic brain inflammation. Neurobiol. Dis..

[83] De Silva T.M., Brait V.H., Drummond G.R., Sobey C.G., Miller A.A. (2011). NOX2 oxidase activity accounts for the oxidative stress and vasomotor dysfunction in mouse cerebral arteries following ischemic stroke. PLoS One.

[84] Kleinschnitz C., Grund H., Wingler K., Armitage M.E., Jones E., Mittal M., Barit D., Schwarz T., Geis C., Kraft P. (2010). Post-stroke inhibition of induced NADPH oxidase type 4 prevents oxidative stress and neurodegeneration. PLoS Biol..

[85] Suzuki Y., Hattori K., Hamanaka J., Murase T., Egashira Y., Mishiro K., Ishiguro M., Tsuruma K., Hirose Y., Tanaka H. (2012). Pharmacological inhibition of TLR4-NOX4 signal protects against neuronal death in transient focal ischemia. Sci. Rep..

[86] Jackman K.A., Miller A.A., Drummond G.R., Sobey C.G. (2009). Importance of NOX1 for angiotensin II-induced cerebrovascular superoxide production and cortical infarct volume following ischemic stroke. Brain Res..

[87] Kahles T., Kohnen A., Heumueller S., Rappert A., Bechmann I., Liebner S., Wittko I.M., Neumann-Haefelin T., Steinmetz H., Schroeder K. (2010). NADPH oxidase NOX1 contributes to ischemic injury in experimental stroke in mice. Neurobiol. Dis..

[88] Radermacher K.A., Wingler K., Kleikers P., Altenhofer S., Hermans J., Kleinschnitz C., Hhw Schmidt H. (2012). The 1027th target candidate in stroke: Will NADPH oxidase hold up?. Exp. Transl. Stroke Med..

[89] Altenhofer S., Radermacher K.A., Kleikers P., Wingler K., Schmidt H.H. (2014). Evolution of NADPH oxidase inhibitors: Selectivity and mechanisms for target engagement. Antioxid. Redox Signal..

[90] Khan F., George J., Wong K., McSwiggan S., Struthers A.D., Belch J.J. (2008). Allopurinol treatment reduces arterial wave reflection in stroke survivors. Cardiovasc. Ther..

[91] Muir K.W. (2006). Glutamate-based therapeutic approaches: Clinical trials with nmda antagonists. Curr. Opin. Pharmacol..

[92] Dawson J., Quinn T.J., Harrow C., Lees K.R., Walters M.R. (2009). The effect of allopurinol on the cerebral vasculature of patients with subcortical stroke; a randomized trial. Br. J. Clin. Pharmacol..

[93] Park C.K., Hall E.D. (1994). Dose-response analysis of the effect of 21-aminosteroid tirilazad mesylate (U-74006F) upon neurological outcome and ischemic brain damage in permanent focal cerebral ischemia. Brain Res..

[94] Xue D., Slivka A., Buchan A.M. (1992). Tirilazad reduces cortical infarction after transient but not permanent focal cerebral ischemia in rats. Stroke.

[95] Sena E., Wheble P., Sandercock P., Macleod M. (2007). Systematic review and meta-analysis of the efficacy of tirilazad in experimental stroke. Stroke.

[96] RANTTAS (1996). A randomized trial of tirilazad mesylate in patients with acute stroke (ranttas). The ranttas investigators. Stroke.

[97] Fleishaker J.C., Hulst-Pearson L.K., Peters G.R. (1995). Effect of gender and menopausal status on the pharmacokinetics of tirilazad mesylate in healthy subjects. Am. J. Ther..

[98] Kuroda S., Tsuchidate R., Smith M.L., Maples K.R., Siesjo B.K. (1999). Neuroprotective effects of a novel nitrone, NXY-059, after transient focal cerebral ischemia in the rat. J. Cereb. Blood Flow Metab..

[99] Zhao Z., Cheng M., Maples K.R., Ma J.Y., Buchan A.M. (2001). NXY-059, a novel free radical trapping compound, reduces cortical infarction after permanent focal cerebral ischemia in the rat. Brain Res..

[100] Sydserff S.G., Borelli A.R., Green A.R., Cross A.J. (2002). Effect of NXY-059 on infarct volume after transient or permanent middle cerebral artery occlusion in the rat; studies on dose, plasma concentration and therapeutic time window. Br. J. Pharmacol..

[101] Marshall J.W., Duffin K.J., Green A.R., Ridley R.M. (2001). NXY-059, a free radical—Trapping agent, substantially lessens the functional disability resulting from cerebral ischemia in a primate species. Stroke.

[102] Marshall J.W., Cummings R.M., Bowes L.J., Ridley R.M., Green A.R. (2003). Functional and histological evidence for the protective effect of NXY-059 in a primate model of stroke when given 4 h after occlusion. Stroke.

[103] Lees K.R., Davalos A., Davis S.M., Diener H.C., Grotta J., Lyden P., Shuaib A., Ashwood T., Hardemark H.G., Wasiewski W. (2006). Additional outcomes and subgroup analyses of NXY-059 for acute ischemic stroke in the SAINT I trial. Stroke.

[104] Shuaib A., Lees K.R., Lyden P., Grotta J., Davalos A., Davis S.M., Diener H.C., Ashwood T., Wasiewski W.W., Emeribe U. (2007). NXY-059 for the treatment of acute ischemic stroke. N. Engl. J. Med..

[105] Koziol J.A., Feng A.C. (2006). On the analysis and interpretation of outcome measures in stroke clinical trials: Lessons from the saint i study of NXY-059 for acute ischemic stroke. Stroke.

[106] Saver J.L. (2007). Clinical impact of NXY-059 demonstrated in the saint I trial: Derivation of number needed to treat for benefit over entire range of functional disability. Stroke.

[107] Fisher M., Lees K., Papadakis M., Buchan A.M. (2006). NXY-059: Brain or vessel protection. Stroke.

[108] Watanabe T., Tanaka M., Watanabe K., Takamatsu Y., Tobe A. (2004). Research and development of the free radical scavenger edaravone as a neuroprotectant. Yakugaku Zasshi.

[109] Lapchak P.A. (2010). A critical assessment of edaravone acute ischemic stroke efficacy trials: Is edaravone an effective neuroprotective therapy?. Exp. Opin. Pharmacother..

[110] Higashi Y. (2009). Edaravone for the treatment of acute cerebral infarction: Role of endothelium-derived nitric oxide and oxidative stress. Exp. Opin. Pharmacother..

[111] Watanabe T., Yuki S., Egawa M., Nishi H. (1994). Protective effects of MCI-186 on cerebral-ischemia—Possible involvement of free-radical scavenging and antioxidant actions. J. Pharmacol. Exp. Ther..

[112] Wu T.W., Zeng L.H., Wu J., Fung K.P. (2000). MCI-186: Further histochemical and biochemical evidence of neuroprotection. Life Sci..

[113] Jin Y.J., Mima T., Raicu V., Park K.C., Shimizu K. (2002). Combined argatroban and edaravone caused additive neuroprotection against 15 min of forebrain ischemia in gerbils. Neurosci. Res..

[114] Ikeda S., Harada K., Ohwatashi A., Kamikawa Y. (2013). Effects of edaravone, a free radical scavenger, on photochemically induced cerebral infarction in a rat hemiplegic model. Sci. World J..

[115] Otomo E., Tohgi H., Kogure K., Hirai S., Takakura K., Terashi A., Gotoh F., Maruyama S., Tazaki Y., Shinohara Y. (2003). Effect of a novel free radical scavenger, edaravone (MCI-186), on acute brain infarction—Randomized, placebo-controlled, double-blind study at multicenters. Cerebrovas. Dis..

[116] Inatomi Y., Takita T., Yonehara T., Fujioka S., Hashimoto Y., Hirano T., Uchino M. (2006). Efficacy of edaravone in cardioembolic stroke. Int. Med..

[117] Nakase T., Yoshioka S., Suzuki A. (2011). Free radical scavenger, edaravone, reduces the lesion size of lacunar infarction in human brain ischemic stroke. BMC Neurol..

[118] Warner D.S., Sheng H., Batinic-Haberle I. (2004). Oxidants, antioxidants and the ischemic brain. J. Exp. Biol..

[119] Gaspar T., Domoki F., Lenti L., Institoris A., Snipes J.A., Bari F., Busija D.W. (2009). Neuroprotective effect of adenoviral catalase gene transfer in cortical neuronal cultures. Brain Res..

[120] Kim D.W., Jeong H.J., Kang H.W., Shin M.J., Sohn E.J., Kim M.J., Ahn E.H., An J.J., Jang S.H., Yoo K.Y. (2009). Transduced human PEP-1-catalase fusion protein attenuates ischemic neuronal damage. Free Radic. Biol. Med..

[121] Gu W., Zhao H., Yenari M.A., Sapolsky R.M., Steinberg G.K. (2004). Catalase over-expression protects striatal neurons from transient focal cerebral ischemia. Neuroreport.

[122] Weisbrot-Lefkowitz M., Reuhl K., Perry B., Chan P.H., Inouye M., Mirochnitchenko O. (1998). Overexpression of human glutathione peroxidase protects transgenic mice against focal cerebral ischemia/reperfusion damage. Brain Res. Mol. Brain Res..

[123] Ishibashi N., Prokopenko O., Weisbrot-Lefkowitz M., Reuhl K.R., Mirochnitchenko O. (2002). Glutathione peroxidase inhibits cell death and glial activation following experimental stroke. Brain Res. Mol. Brain Res..

[124] Crack P.J., Taylor J.M., Flentjar N.J., de Haan J., Hertzog P., Iannello R.C., Kola I. (2001). Increased infarct size and exacerbated apoptosis in the glutathione peroxidase-1 (GPX-1) knockout mouse brain in response to ischemia/reperfusion injury. J. Neurochem..

[125] Fujimura M., Morita-Fujimura Y., Noshita N., Sugawara T., Kawase M., Chan P.H. (2000). The cytosolic antioxidant copper/zinc-superoxide dismutase prevents the early release of mitochondrial cytochrome c in ischemic brain after transient focal cerebral ischemia in mice. J. Neurosci..

[126] Kondo T., Reaume A.G., Huang T.T., Murakami K., Carlson E., Chen S., Scott R.W., Epstein C.J., Chan P.H. (1997). Edema formation exacerbates neurological and histological outcomes after focal cerebral ischemia in cuzn-superoxide dismutase gene knockout mutant mice. Brain Edema X.

[127] Davis A.S., Zhao H., Sun G.H., Sapolsky R.M., Steinberg G.K. (2007). Gene therapy using SOD1 protects striatal neurons from experimental stroke. Neurosci. Lett..

[128] Sheng H., Bart R.D., Oury T.D., Pearlstein R.D., Crapo J.D., Warner D.S. (1999). Mice overexpressing extracellular superoxide dismutase have increased resistance to focal cerebral ischemia. Neuroscience.

[129] Li P.F. (1995). Oxidative modification of cupro-zinc superoxide dismutase by reactive oxygen species. Sheng Li Ke Xue Jin Zhan.

[130] Kim G.W., Chan P.H. (2002). Involvement of superoxide in excitotoxicity and DNA fragmentation in striatal vulnerability in mice after treatment with the mitochondrial toxin, 3-nitropropionic acid. J. Cereb. Blood Flow Metab..

[131] Maier C.M., Hsieh L., Crandall T., Narasinnhan P., Chan P.H. (2006). A new approach for the investigation of reperfusion-related brain injury. Biochem. Soc. Trans..

[132] Jung J.E., Kim G.S., Narasimhan P., Chan P.H. (2009). STAT3 regulates the transcription of the mouse Mn-SOD gene as a neuroprotectant in cerebral ischemic reperfusion. J. Cereb. Blood Flow Metab..

[133] Jung J.E., Kim G.S., Chan P.H. (2011). Neuroprotection by interleukin-6 is mediated by signal transducer and activator of transcription 3 and antioxidative signaling in ischemic stroke. Stroke.

[134] Namura S., Nagata I., Takami S., Masayasu H., Kikuchi H. (2001). Ebselen reduces cytochrome c release from mitochondria and subsequent DNA fragmentation after transient focal cerebral ischemia in mice. Stroke.

[135] Imai H., Masayasu H., Dewar D., Graham D.I., Macrae I.M. (2001). Ebselen protects both gray and white matter in a rodent model of focal cerebral ischemia. Stroke.

[136] Takasago T., Peters E.E., Graham D.I., Masayasu H., Macrae I.M. (1997). Neuroprotective efficacy of ebselen, an anti-oxidant with anti-inflammatory actions, in a rodent model of permanent middle cerebral artery occlusion. Br. J. Pharmacol..

[137] Yamaguchi T., Sano K., Takakura K., Saito I., Shinohara Y., Asano T., Yasuhara H. (1998). Ebselen in acute ischemic stroke: A placebo-controlled, double-blind clinical trial. Ebselen study group. Stroke.

[138] Ohsawa I., Ishikawa M., Takahashi K., Watanabe M., Nishimaki K., Yamagata K., Katsura K.-I., Katayama Y., Asoh S., Ohta S. (2007). Hydrogen acts as a therapeutic antioxidant by selectively reducing cytotoxic oxygen radicals. Nat. Med..

[139] Singhal A.B., Wang X., Sumii T., Mori T., Lo E.H. (2002). Effects of normobaric hyperoxia in a rat model of focal cerebral ischemia-reperfusion. J. Cereb. Blood Flow Metab..

[140] Singhal A.B., Dijkhuizen R.M., Rosen B.R., Lo E.H. (2002). Normobaric hyperoxia reduces MRI diffusion abnormalities and infarct size in experimental stroke. Neurology.

[141] Esposito E., Mandeville E.T., Hayakawa K., Singhal A.B., Lo E.H. (2013). Effects of normobaric oxygen on the progression of focal cerebral ischemia in rats. Exp. Neurol..

[142] Kim H.Y., Singhal A.B., Lo E.H. (2005). Normobaric hyperoxia extends the reperfusion window in focal cerebral ischemia. Ann. Neurol..

[143] Geng X., Fu P., Ji X., Peng C., Fredrickson V., Sy C., Meng R., Ling F., Du H., Tan X. (2013). Synergetic neuroprotection of normobaric oxygenation and ethanol in ischemic stroke through improved oxidative mechanism. Stroke.

[144] Geng X., Parmar S., Li X., Peng C., Ji X., Chakraborty T., Li W.A., Du H., Tan X., Ling F. (2013). Reduced apoptosis by combining normobaric oxygenation with ethanol in transient ischemic stroke. Brain Res..

[145] Dalkara T., Moskowitz M.A. (1994). The complex role of nitric oxide in the pathophysiology of focal cerebral ischemia. Brain Pathol..

[146] Dalkara T., Yoshida T., Irikura K., Moskowitz M.A. (1994). Dual role of nitric oxide in focal cerebral ischemia. Neuropharmacology.

[147] Huang Y., McNamara J.O. (2004). Ischemic stroke: Acidotoxicity is a perpetrator. Cell.

[148] Charriaut-Marlangue C., Bonnin P., Gharib A., Leger P.-L., Villapol S., Pocard M., Gressens P., Renolleau S., Baud O. (2012). Inhaled nitric oxide reduces brain damage by collateral recruitment in a neonatal stroke model. Stroke.

[149] Terpolilli N.A., Kim S.-W., Thal S.C., Kataoka H., Zeisig V., Nitzsche B., Klaesner B., Zhu C., Schwarzmaier S., Meissner L. (2012). Inhalation of nitric oxide prevents ischemic brain damage in experimental stroke by selective dilatation of collateral arterioles. Circ. Res..

[150] Lesage A.S., Peeters L., Leysen J.E. (1996). Lubeluzole, a novel long-term neuroprotectant, inhibits the glutamate-activated nitric oxide synthase pathway. J. Pharmacol. Exp. Ther..

[151] Ashton D., Willems R., Wynants J., van Reempts J., Marrannes R., Clincke G. (1997). Altered Na^+^-channel function as an *in vitro* model of the ischemic penumbra: Action of lubeluzole and other neuroprotective drugs. Brain Res..

[152] Maiese K., TenBroeke M., Kue I. (1997). Neuroprotection of lubeluzole is mediated through the signal transduction pathways of nitric oxide. J. Neurochem..

[153] De Ryck M., Keersmaekers R., Duytschaever H., Claes C., Clincke G., Janssen M., van Reet G. (1996). Lubeluzole protects sensorimotor function and reduces infarct size in a photochemical stroke model in rats. J. Pharmacol. Exp. Ther..

[154] Aronowski J., Strong R., Grotta J.C. (1996). Treatment of experimental focal ischemia in rats with lubeluzole. Neuropharmacology.

[155] Diener H.C., Hacke W., Hennerici M., Radberg J., Hantson L., de Keyser J. (1996). Lubeluzole in acute ischemic stroke. A double-blind, placebo-controlled phase II trial. Lubeluzole international study group. Stroke.

[156] Grotta J. (1997). Lubeluzole treatment of acute ischemic stroke. The US and Canadian lubeluzole ischemic stroke study group. Stroke.

[157] Diener H.C. (1998). Multinational randomised controlled trial of lubeluzole in acute ischaemic stroke. European and Australian lubeluzole ischaemic stroke study group. Cerebrovasc. Dis.

[158] Diener H.C., Cortens M., Ford G., Grotta J., Hacke W., Kaste M., Koudstaal P.J., Wessel T. (2000). Lubeluzole in acute ischemic stroke treatment: A double-blind study with an 8-h inclusion window comparing a 10-mg daily dose of lubeluzole with placebo. Stroke.

[159] Gandolfo C., Sandercock P., Conti M. (2002). Lubeluzole for acute ischaemic stroke. Cochrane Database Syst. Rev..

[160] Weisiger R.A., Fridovich I. (1973). Mitochondrial superoxide simutase. Site of synthesis and intramitochondrial localization. J. Biol. Chem..

[161] Abramov A.Y., Scorziello A., Duchen M.R. (2007). Three distinct mechanisms generate oxygen free radicals in neurons and contribute to cell death during anoxia and reoxygenation. J. Neurosci..

[162] Murphy M.P. (2014). Antioxidants as therapies: Can we improve on nature?. Free Radic. Biol. Med..

[163] Siler-Marsiglio K.I., Pan Q., Paiva M., Madorsky F., Khurana N.C., Heaton M.B. (2005). Mitochondrially targeted vitamin E and vitamin E mitigate ethanol-mediated effects on cerebellar granule cell antioxidant defense systems. Brain Res..

[164] Dhanasekaran A., Kotamraju S., Kalivendi S.V., Matsunaga T., Shang T., Keszler A., Joseph J., Kalyanaraman B. (2004). Supplementation of endothelial cells with mitochondria-targeted antioxidants inhibit peroxide-induced mitochondrial iron uptake, oxidative damage, and apoptosis. J. Biol. Chem..

[165] Murphy M.P., Smith R.A. (2007). Targeting antioxidants to mitochondria by conjugation to lipophilic cations. Annu. Rev. Pharmacol. Toxicol..

[166] Kelso G.F., Porteous C.M., Hughes G., Ledgerwood E.C., Gane A.M., Smith R.A.J., Murphy M.P. (2002). Prevention of mitochondrial oxidative damage using targeted antioxidants. Increasing Healthy Life Span.

[167] Smith R.A., Murphy M.P. (2010). Animal and human studies with the mitochondria-targeted antioxidant mitoq. Ann. N. Y. Acad.Sci..

[168] Adlam V.J., Harrison J.C., Porteous C.M., James A.M., Smith R.A., Murphy M.P., Sammut I.A. (2005). Targeting an antioxidant to mitochondria decreases cardiac ischemia-reperfusion injury. FASEB J..

[169] Graham D., Huynh N.N., Hamilton C.A., Beattie E., Smith R.A., Cocheme H.M., Murphy M.P., Dominiczak A.F. (2009). Mitochondria-targeted antioxidant MitoQ10 improves endothelial function and attenuates cardiac hypertrophy. Hypertension.

[170] McLachlan J., Beattie E., Murphy M.P., Koh-Tan C.H., Olson E., Beattie W., Dominiczak A.F., Nicklin S.A., Graham D. (2014). Combined therapeutic benefit of mitochondria-targeted antioxidant, MitoQ10, and angiotensin receptor blocker, losartan, on cardiovascular function. J. Hypertens..

[171] Wani W.Y., Gudup S., Sunkaria A., Bal A., Singh P.P., Kandimalla R.J., Sharma D.R., Gill K.D. (2011). Protective efficacy of mitochondrial targeted antioxidant mitoq against dichlorvos induced oxidative stress and cell death in rat brain. Neuropharmacology.

[172] Hobbs C.E., Murphy M.P., Smith R.A., Oorschot D.E. (2008). Neonatal rat hypoxia-ischemia: Effect of the anti-oxidant mitoquinol, and S-PBN. Pediatr. Int..

[173] Miura S., Ishida-Nakajima W., Ishida A., Kawamura M., Ohmura A., Oguma R., Sato Y., Takahashi T. (2009). Ascorbic acid protects the newborn rat brain from hypoxic-ischemia. Brain Dev..

[174] Ducruet A.F., Mack W.J., Mocco J., Hoh D.J., Coon A.L., D’Ambrosio A.L., Winfree C.J., Pinsky D.J., Connolly E.S. (2011). Preclinical evaluation of postischemic dehydroascorbic acid administration in a large-animal stroke model. Transl. Stroke Res..

[175] Zhang X.H., Lei H., Liu A.J., Zou Y.X., Shen F.M., Su D.F. (2011). Increased oxidative stress is responsible for severer cerebral infarction in stroke-prone spontaneously hypertensive rats. CNS Neurosci. Ther..

[176] Yokoyama T., Date C., Kokubo Y., Yoshiike N., Matsumura Y., Tanaka H. (2000). Serum vitamin C concentration was inversely associated with subsequent 20-year incidence of stroke in a japanese rural community. The shibata study. Stroke.

[177] Myint P.K., Luben R.N., Welch A.A., Bingham S.A., Wareham N.J., Khaw K.T. (2008). Plasma vitamin C concentrations predict risk of incident stroke over 10 year in 20,649 participants of the european prospective investigation into cancer norfolk prospective population study. Am. J. Clin. Nutr..

[178] Kubota Y., Iso H., Date C., Kikuchi S., Watanabe Y., Wada Y., Inaba Y., Tamakoshi A. (2011). Dietary intakes of antioxidant vitamins and mortality from cardiovascular disease: The Japan collaborative cohort study (JACC) study. Stroke.

[179] Heart Protection Study Collaborative Group (2002). MRC/BHF heart protection study of antioxidant vitamin supplementation in 20,536 high-risk individuals: A randomised placebo-controlled trial. Lancet.

[180] Cook N.R., Albert C.M., Gaziano J.M., Zaharris E., MacFadyen J., Danielson E., Buring J.E., Manson J.E. (2007). A randomized factorial trial of vitamins C and E and beta carotene in the secondary prevention of cardiovascular events in women: Results from the women’s antioxidant cardiovascular study. Arch. Intern. Med..

[181] Sesso H.D., Buring J.E., Christen W.G., Kurth T., Belanger C., MacFadyen J., Bubes V., Manson J.E., Glynn R.J., Gaziano J.M. (2008). Vitamins E and C in the prevention of cardiovascular disease in men: The physicians’ health study II randomized controlled trial. JAMA.

[182] Schurks M., Glynn R.J., Rist P.M., Tzourio C., Kurth T. (2010). Effects of vitamin E on stroke subtypes: Meta-analysis of randomised controlled trials. Br. Med. J..

[183] Bin Q., Hu X.Y., Cao Y., Gao F. (2011). The role of vitamin E (tocopherol) supplementation in the prevention of stroke a meta-analysis of 13 randomised controlled trials. Thromb. Haemost..

[184] Wang G.L., Semenza G.L. (1995). Purification and characterization of hypoxia-inducible factor 1. J. Biol. Chem..

[185] Bernaudin M., Nedelec A.S., Divoux D., MacKenzie E.T., Petit E., Schumann-Bard P. (2002). Normobaric hypoxia induces tolerance to focal permanent cerebral ischemia in association with an increased expression of hypoxia-inducible factor-1 and its target genes, erythropoietin and VEGF, in the adult mouse brain. J. Cereb. Blood Flow Metab..

[186] Jones N.M., Bergeron M. (2001). Hypoxic preconditioning induces changes in HIF-1 target genes in neonatal rat brain. J. Cereb. Blood Flow Metab..

[187] Prass K., Ruscher K., Karsch M., Isaev N., Megow D., Priller J., Scharff A., Dirnagl U., Meisel A. (2002). Desferrioxamine induces delayed tolerance against cerebral ischemia *in vivo* and *in vitro*. J. Cereb. Blood Flow Metab..

[188] Freret T., Valable S., Chazalviel L., Saulnier R., Mackenzie E.T., Petit E., Bernaudin M., Boulouard M., Schumann-Bard P. (2006). Delayed administration of deferoxamine reduces brain damage and promotes functional recovery after transient focal cerebral ischemia in the rat. Eur. J. Neurosci..

[189] Wang X., Liu J., Zhu H., Tejima E., Tsuji K., Murata Y., Atochin D.N., Huang P.L., Zhang C., Lo E.H. (2008). Effects of neuroglobin overexpression on acute brain injury and long-term outcomes after focal cerebral ischemia. Stroke.

[190] Li R.C., Guo S.Z., Lee S.K., Gozal D. (2010). Neuroglobin protects neurons against oxidative stress in global ischemia. J. Cereb. Blood Flow Metab..

[191] Sun Y., Jin K., Peel A., Mao X.O., Xie L., Greenberg D.A. (2003). Neuroglobin protects the brain from experimental stroke *in vivo*. Proc. Natl. Acad. Sci. USA.

[192] Cai B., Lin Y., Xue X.H., Fang L., Wang N., Wu Z.Y. (2011). Tat-mediated delivery of neuroglobin protects against focal cerebral ischemia in mice. Exp. Neurol..

[193] Ord E.N., Shirley R., McClure J.D., McCabe C., Kremer E.J., Macrae I.M., Work L.M. (2013). Combined antiapoptotic and antioxidant approach to acute neuroprotection for stroke in hypertensive rats. J. Cereb. Blood Flow Metab..

[194] Hacke W., Kaste M., Bluhmki E., Brozman M., Davalos A., Guidetti D., Larrue V., Lees K.R., Medeghri Z., Machnig T. (2008). Thrombolysis with alteplase 3 to 4.5 h after acute ischemic stroke. N. Engl. J. Med..

[195] Liu D., Cheng T., Guo H., Fernandez J.A., Griffin J.H., Song X., Zlokovic B.V. (2004). Tissue plasminogen activator neurovascular toxicity is controlled by activated protein c. Nat. Med..

[196] Baker A.H., Sica V., Work L.M., Williams-Ignarro S., de Nigris F., Lerman L.O., Casamassimi A., Lanza A., Schiano C., Rienzo M. (2007). Brain protection using autologous bone marrow cell, metalloproteinase inhibitors, and metabolic treatment in cerebral ischemia. Proc. Natl. Acad. Sci. USA.

[197] Asahi M., Asahi K., Wang X., Lo E.H. (2000). Reduction of tissue plasminogen activator-induced hemorrhage and brain injury by free radical spin trapping after embolic focal cerebral ischemia in rats. J. Cereb. Blood Flow Metab..

[198] Barth A., Barth L., Newell D.W. (1996). Combination therapy with MK-801 and alpha-phenyl-tert-butyl-nitrone enhances protection against ischemic neuronal damage in organotypic hippocampal slice cultures. Exp. Neurol..

[199] Deguchi K., Miyazaki K., Tian F., Liu N., Liu W., Kawai H., Omote Y., Kono S., Yunoki T., Deguchi S. (2012). Modifying neurorepair and neuroregenerative factors with TPA and edaravone after transient middle cerebral artery occlusion in rat brain. Brain Res..

[200] David H.N., Haelewyn B., Degoulet M., Colomb D.G., Risso J.J., Abraini J.H. (2012). Prothrombolytic action of normobaric oxygen given alone or in combination with recombinant tissue-plasminogen activator in a rat model of thromboembolic stroke. J. Appl. Physiol..

[201] Schmid-Elsaesser R., Hungerhuber E., Zausinger S., Baethmann A., Reulen H.J. (1999). Neuroprotective efficacy of combination therapy with two different antioxidants in rats subjected to transient focal ischemia. Brain Res..

[202] O’Collins V.E., Macleod M.R., Donnan G.A., Howells D.W. (2012). Evaluation of combination therapy in animal models of cerebral ischemia. J. Cereb. Blood Flow Metab..

[203] Dirnagl U. (2006). Bench to bedside: The quest for quality in experimental stroke research. J. Cereb. Blood Flow Metab..

[204] Stroke Therapy Academic Industry Roundtable (STAIR) (1999). Recommendations for standards regarding preclinical neuroprotective and restorative drug development. Stroke.

[205] Fisher M., Feuerstein G., Howells D.W., Hurn P.D., Kent T.A., Savitz S.I., Lo E.H., Group F.T.S. (2009). Update of the stroke therapy academic industry roundtable preclinical recommendations. Stroke.

[206] O’Collins V.E., Macleod M.R., Donnan G.A., Horky L.L., van der Worp B.H., Howells D.W. (2006). 1026 Experimental treatments in acute stroke. Ann. Neurol..

[207] Sena E.S., van der Worp H.B., Howells D., Macleod M. (2007). How can we improve the pre-clinical development of drugs for stroke?. Trends Neurosci..

[208] O’Collins V.E., Donnan G.A., Macleod M.R., Howells D.W. (2009). Scope of preclinical testing *versus* quality control within experiments. Stroke.

[209] Macleod M.R., Fisher M., O’Collins V., Sena E.S., Dirnagl U., Bath P.M.W., Buchan A., van der Worp H.B., Traystman R., Minematsu K. (2009). Good laboratory practice: Preventing introduction of bias at the bench. Stroke.

[210] Kilkenny C., Browne W.J., Cuthill I.C., Emerson M., Altman D.G. (2010). Improving bioscience research reporting: The arrive guidelines for reporting animal research. PLoS Biol..

[211] Dirnagl U., Hakim A., Macleod M., Fisher M., Howells D., Alan S.M., Steinberg G., Planas A., Boltze J., Savitz S. (2013). A concerted appeal for international cooperation in preclinical stroke research. Stroke.

